# Upregulation of interferon-γ response genes in monocytes and T cells identified by single-cell transcriptomics in patients with anti-citrullinated peptide antibody-positive early rheumatoid arthritis

**DOI:** 10.3389/fimmu.2024.1439082

**Published:** 2025-01-14

**Authors:** Bong-Ki Hong, Sungyong You, Jung Gon Kim, Minhyung Kim, Naeun Lee, Kijun Lee, In-Pyo Baek, Ji Hyeon Ju, Wan-Uk Kim, Ho-Youn Kim

**Affiliations:** ^1^ Center for Integrative Rheumatoid Transcriptomics and Dynamics, The Catholic University of Korea, Seoul, Republic of Korea; ^2^ Urology and Computational Biomedicine, Cedars-Sinai Medical Center, Los Angeles, CA, United States; ^3^ Division of Rheumatology, Department of Internal Medicine, Inje University Ilsan Paik Hospital, Goyang, Republic of Korea; ^4^ Catholic iPSC Research Center, College of Medicine, The Catholic University of Korea, Seoul, Republic of Korea; ^5^ YiPSCELL, Inc., Seoul, Republic of Korea; ^6^ Department of Internal Medicine, The Catholic University of Korea, Seoul St. Mary’s Hospital, Seoul, Republic of Korea; ^7^ The Catholic University of Korea and Ho-Youn Kim’s Clinic for Arthritis Rheumatism, Seoul, Republic of Korea

**Keywords:** single-cell transcriptomics, peripheral blood mononuclear cells, anti-citrullinated peptide antibody, rheumatoid arthritis, rheumatoid arthritis pathogenesis, Th1 immunity, interferon signature, IFITM2/3

## Abstract

**Introduction:**

Our aim was to investigate the insufficiently understood differences in the immune system between anti-citrullinated peptide antibody (ACPA)-positive (ACPA^+^) and ACPA-negative (ACPA^-^) early rheumatoid arthritis (eRA) patients.

**Methods:**

We performed multiple cytokine assays using sera from drug-naïve ACPA^+^ and ACPA^-^ eRA patients. Additionally, we conducted single-cell RNA sequencing of CD45^+^ cells from peripheral blood samples to analyze and compare the distribution and functional characteristics of the cell subsets based on the ACPA status.

**Results:**

Serum concentrations of interferon-γ (IFN-γ) and interleukin (IL)-12 were higher in ACPA^+^ eRA than in ACPA^-^ eRA. Single-cell transcriptome analysis of 37,318 cells identified 17 distinct cell types and revealed the expansion of IL1B^+^ proinflammatory monocytes, IL7R^+^ T cells, and CD8^+^ CCL4^+^ T cells in ACPA^+^ eRA. Furthermore, we observed an enrichment of IFN-γ response genes in nearly all monocytes and T cells of ACPA^+^ eRA subsets. Heightened interactions between IFN-γ and IFN-γ receptors were observed in ACPA^+^ eRA, particularly between monocytes and T cells. We examined *IFITM2* and *IFITM3* as potential key markers in ACPA^+^ eRA given their pronounced upregulation and association with the IFN response. Specifically, the expression of these genes was elevated in IL1B^+^ proinflammatory monocytes (likely M1 monocytes), correlating with serum IFN-γ levels.

**Discussion:**

Compared to ACPA^-^ eRA, ACPA^+^ eRA showed higher serum IFN-γ and IL-12 levels, upregulated IFN-γ response genes, and enhanced IFN-γ-driven monocyte-T cell interactions. These distinct immune features of the peripheral circulation in ACPA^+^ eRA suggest a role for type 1 helper T cell-related immunity in its pathogenesis.

## Introduction

Rheumatoid arthritis (RA) is a chronic autoimmune disease characterized by inflammation of the joint synovium (1). It results from complex interplays of synovial T cells, B cells, macrophages, dendritic cells (DCs), and fibroblast-like synoviocytes (FLSs) leading to joint destruction via autoreactive antibodies, chemokines, and pathogenic cytokines (2). Over recent decades, targeted biologics against lymphocytes and key pathogenic cytokines have achieved great success in managing RA (1). Nevertheless, 6% of patients with RA in Japan and 10% in the United Kingdom are refractory to these therapies, highlighting the limitations of the current treatment strategies (3, 4).

RA is a heterogeneous disease with various endo-phenotypes, for which personalized medicine is desirable (5, 6). Although a personalized approach has not been established in RA, there have been efforts to guide therapy using the anti-citrullinated peptide antibody (ACPA), the most commonly used diagnostic and prognostic biomarker (7). Clinical studies have suggested a better response to Abatacept than a tumor necrosis factor inhibitor in ACPA^+^ RA (8). Moreover, longer drug retention of a Janus kinase inhibitor (JAK) was observed in ACPA^+^ RA than in ACPA^-^ RA (9). Together, these earlier reports indicate that the presence or absence of ACPA could significantly shape the most effective treatment strategy for RA, underscoring the importance of a patient-centric approach to RA treatment by considering each patient’s ACPA status.

To utilize ACPA as a biomarker in guiding treatment, it is crucial to comprehend the immunologic difference according to the presence of ACPA. Previously known, immune complexes of ACPAs and citrullinated peptides can promote pro-inflammatory reactions of macrophages through binding to Fc receptors (10). Antibodies against mutated citrullinated vimentin, a highly specific ACPA for RA, can activate osteoclastogenesis and bone resorption (11). Transcriptome analysis has revealed that chemokine profiles of myeloid cells are altered and cytotoxic properties of T cells are differentially upregulated in ACPA^+^ RA (12). Despite previous investigations, we have insufficient understanding of the differentiating immunologic characteristics between endotypes of ACPA^+^ and ACPA^-^ RA.

Interferon-gamma (IFN-γ) holds significant interest in the field of autoimmunity research due to its influential role in promoting and regulating inflammation (13). This aspect is particularly critical in the context of RA, where the dominant pathogenic cells are type 1 helper T (Th1) cells, known for their primary production of IFN-γ (2, 13, 14). A number of studies have documented elevated levels of IFN-γ in patients with RA, as well as in mouse models of autoimmune arthritis (15–17). JAK inhibitor targeting the IFN pathway, as well as other pathogenic cytokines, have shown excellent efficacy in the treatment of RA (18). Considering the highly heterogeneous nature of RA, an in-depth understanding of the level of IFN-γ expression in individual RA patients is needed.

Here, to gain insight into the immunological background for tailored medicine, we aimed to comparatively study immunologic characteristics according to ACPA status. To this end, we performed multiplex cytokine assay (MCA) demonstrating increased serum levels of Th1 cell-related cytokines, specifically IL-12 and IFN-γ, in ACPA^+^ early RA (eRA). Subsequently, we performed single-cell RNA sequencing (scRNA-seq) analysis of peripheral blood mononuclear cells (PBMCs) from eRA patients and then compared gene expression and cell-cell interaction patterns between ACPA^+^ and ACPA^-^ eRA. It revealed that interferon response genes (IRGs), particularly *IFITM2* and *IFITM3*, were distinctly upregulated in monocytes and T cells of ACPA^+^ eRA compared to those of ACPA^-^ eRA. Such upregulation in ACPA^+^ eRA might have resulted from Th1-skewed antigen-specific T-cell immunity and its related activation of monocytes involved in RA. Furthermore, we found a positive correlation between expression levels of the major IRGs in monocytes and T cells and levels of serum IL-6 and IFN-γ. Collectively, these findings provide novel insights into the immuno-pathogenic mechanisms underlying RA, potentially contributing to the development of more effective, personalized treatments for this complex disease.

## Materials and methods

### Patient recruitment and sample processing

Untreated (no current or prior use of glucocorticoids or disease-modifying anti-rheumatic drugs) patients with early and active RA who met the 2010 ACR/EULAR RA classification criteria (19) were recruited from Seoul St. Mary’s Hospital in Korea. Unclassified arthritis patients were recruited based on the following inclusion criteria: (1) at least one swollen joint in the wrists or hands; (2) negative result for ACPA; (3) symptom duration of less than 12 months. The exclusion criteria for unclassified arthritis were: (1) meeting the 2010 ACR/EULAR RA classification criteria (19); (2) presence of other connective tissue diseases; (3) acute trauma; and (4) current or previous use of glucocorticoids or disease-modifying anti-rheumatic drugs (20, 21). Healthy volunteers were also recruited as controls. Peripheral blood samples were obtained from the participants for scRNA-seq and cytokine assay at the time of recruitment. Patient information, including demographic profile, laboratory markers, and disease activity scores, was collected at the time of blood sampling (**Supplementary Table S1**). Peripheral blood mononuclear cells were isolated using Ficoll-Paque gradient centrifugation. Cell quantity and viability were then determined by Trypan Blue staining. This study was approved by the Institutional Review Board of Seoul St Mary’s Hospital (approval number: KC14TIMI0697). All participants provided written informed consent.

### Multiplex cytokine assay

Concentrations of IFN-γ, IL-12 and IL-6, in serum samples of eRA patients were measured from using Millipore’s MILLIPLEX MAP High Sensitivity Human Cytokine multiplex kit (cat. no. HSTCMAG-28SK; Merck, Billerica, MA, USA) according to the manufacturer’s instructions. The minimum detection limits for the MCA were established at 0.61 pg/mL for IFN-γ, 0.49 pg/mL for IL-12, and 0.18 pg/mL for IL-6.

### Single cell preparation and multiplexing individual samples for scRNA-seq

Cell stocks were thawed in 37°C 10% FBS/DMEM. The samples were washed twice with cold, Ca^2+^- and Mg^2+^-free 0.04% BSA/PBS at 300 × g for 5 min at 4°C. They were then gently resuspended in cold staining buffer (BD Biosciences, catalog no. 554656) and counted using a LUNA-FX7 Automated Fluorescence Cell Counter (Logos Biosystems) with AO/PI staining. To multiplex the samples, each sample was tagged with an antibody-polyadenylated DNA barcode specific for human cells (BD Biosciences, catalog no. 633781). Briefly, the cells were stained with the multiplexing antibody for 20 min at room temperature, followed by three washes with staining buffer (BD Biosciences, catalog no. 554656). After the final wash, the samples were gently resuspended in cold Sample Buffer (BD Biosciences, catalog no. 664887), counted using a LUNA-FX7 Automated Fluorescence Cell Counter (Logos Biosystems), and pooled.

### Single cell capture and cDNA synthesis

Single-cell capture was performed using a BD Rhapsody Express instrument according to the manufacturer’s instructions (BD Biosciences). Briefly, pooled cells from each sample were suspended in cold sample buffer and loaded into a BD Rhapsody cartridge (BD Biosciences, catalog no. 633731). After cell separation, cell-barcode magnetic beads were added to the cartridge. The cells were then lysed and the mRNA capture beads were retrieved. cDNA synthesis and Exonuclease I treatment were performed on the mRNA capture beads using a BD Rhapsody cDNA Kit (BD Biosciences, catalog no. 633773).

### Library preparation and scRNA-seq

According to the ‘mRNA Whole Transcriptome Analysis (WTA) and Sample Tag Library Preparation’ protocol, scRNA-seq libraries were constructed using the BD Rhapsody WTA amplification kit (BD Biosciences, catalog no. 633801). For the WTA library, cDNA was sequentially subjected to random priming and extension (RPE), RPE amplification, and index PCR. For the sample tag library, cDNA was sequentially subjected to nested PCR (PCR 1 and PCR 2) and index PCR. The purified WTA and sample tag libraries were quantified using qPCR according to the qPCR Quantification Protocol Guide (KAPA) and assessed using the 4200 TapeStation System (Agilent Technologies, catalog no. 5067-4626). The libraries were sequenced using the HiSeq platform (Illumina).

### Preprocessing of sequencing data

The raw sequencing data were processed using the BD Rhapsody WTA Analysis Pipeline v1.8 (BD Biosciences) and aligned against the human reference genome (GRCh38) obtained from the Ensembl database. The resulting gene expression matrices were converted to individual Seurat objects using the Seurat package in R (v3.8.0) (22). For each object, we filtered data based on the number of unique molecular identifiers (UMIs) and the number of genes detected. The genes that were expressed in at least five cells, and cells with gene detection between 500 to 2000 were retained. The filtered objects were normalized and their variance stabilized using the SCTransform function of Seurat. We reduced batch effects and performed combined analysis by integrating individual Seurat objects from various batches using the FindIntegrationAnchors and IntegrateData functions in Seurat. We addressed batch effects through a confirmation process as shown in **Supplementary Figure S1**.

### Dimension reduction and major cell type annotation

The number of UMIs, percentage of mitochondrial genes, and cell cycle genes were regressed out, and genes were scaled to unit variance. Principle component analysis (PCA) was performed. Clusters were then identified using UMAP. Cell identity was assigned using known cell markers shown in **Supplementary Figure S2**. We compared gene expression levels between cells in the cluster and those in all the other clusters to determine cluster marker genes. Clusters were manually annotated based on known marker genes. Thereafter, we validated annotations by referring to results from “seurat_annotation,” “human cell atlas,” and “Z_annotation” (**Supplementary Table S2**) (23). Adjacent clusters were merged if they were regarded as identical entities according to the similarity of transcriptomes.

### Detection of differentially expressed genes and pathway analysis

Differential gene expression testing was performed using the ‘FindMarkers’ function within Seurat, employing the Wilcoxon test. All *p*-values were adjusted using Bonferroni correction. Differentially expressed genes (DEGs) were filtered using a minimum log2(fold change) of 0.5 and a maximum adjusted *p*-value of 0.05. They were then ranked by average log2(fold change) and false discovery rate (FDR). Enrichment analysis for functions of the DEGs was conducted using the clusterProfiler package and DAVID (https://david.ncifcrf.gov/) (24). Gene sets were based on Gene Ontology terms and Kyoto Encyclopedia of Genes and Genomes (KEGG) pathways.

### Analysis of cell-cell interaction

To comprehensively analyze cell-to-cell interactions between immune cells, we used SingleCellSignalR (25). We derived potential ligand-receptor interactions based on the expression of a receptor by one cell subpopulation and ligand expression by another. We separately fetched normalized counts from healthy controls, ACPA^+^, and ACPA^-^ eRA patients and used them as input for the algorithm. To validate the cell-to-cell interactions and ligand-receptor interaction result from SinglCellSignalR, we performed the same analysis with CellChat (v.1.0) (26) and CellphoneDB (v.4.0) (27).

### Inferring differentially expressed transcription factors

To determine the relationship between IFN signaling activity and anti-CCP antibodies, we used transcriptomics data to estimate the overall expression of IFN signaling genes for each sample. The decoupleR (v1.6.0) ‘wmean’ method and ‘SCTransform’ normalized data were used to calculate normalized gene expression levels of IFNA, IFNG, IFNAR1, IFANR2, IFNGR1, and IFNGR2 per cell for each unfiltered slide (28).

### Chromatin binding profiles

We searched for chromatin binding sites of IFITM2 and IFITM3 based on chromatin binding profiles provided by ReMap 2022. Detected transcription factors (TFs), which were matched to upregulated TFs derived from master regulator analysis, were visualized with Integrative Genomics Viewer (IGV) (29).

### Data visualization

All plots were generated using the ggplot2 (v3.2.1), pheatmap (v1.0.12), and EnhancedVolcano (v1.2.0) packages in R v4.0.0. Box plots are defined as follows: the middle line corresponds to the median; lower and upper hinges correspond to the first and third quartiles, respectively; the upper whisker extends from the hinge to the largest value, reaching no more than 1.5× the interquartile range (or the distance between the first and third quartiles) from the hinge; and the lower whisker extends from the hinge to the smallest value, not exceeding 1.5× the interquartile range from the hinge. Data beyond the end of whiskers were designated as “outliers”. They were plotted individually.

## Results

### Serum cytokine profiles of ACPA^+^ and ACPA^-^ eRA

**Figure 1:**
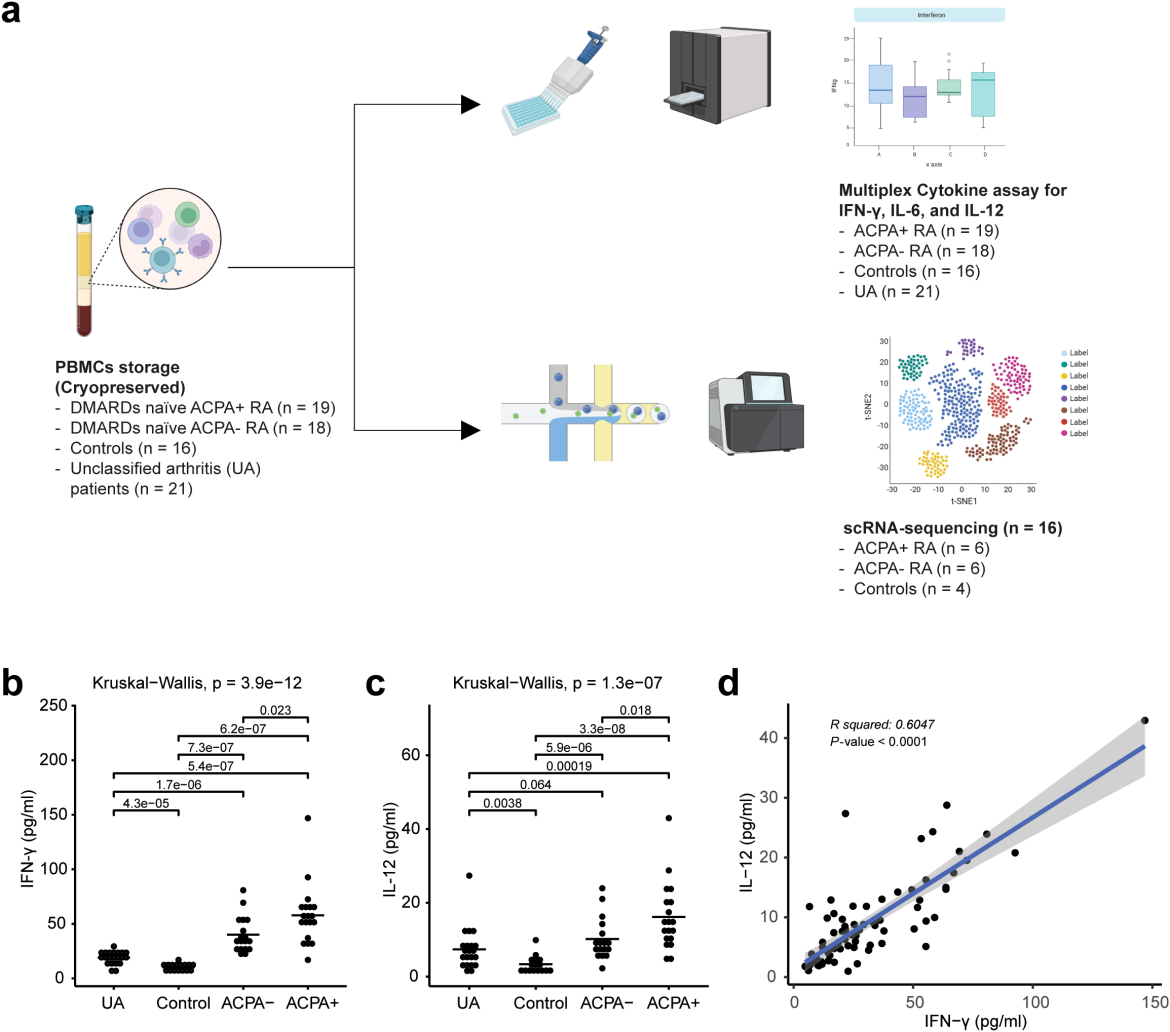
Overall study design and multiplex cytokine analysis of rheumatoid arthritis (RA) peripheral blood mononuclear cells (PBMCs). **(A)** Workflow chart outlining the overall study design, illustrating each step from patient selection to data analysis, and highlighting the methodologies used in the extraction and analysis of PBMCs from unclassified arthritis (UA), healthy controls, ACPA-negative early RA (ACPA^-^ eRA), and ACPA-positive early RA (ACPA^+^ eRA). **(B)** Dot plot showing the serum concentration levels of interferon-γ (IFN-γ) in UA (n = 21), controls (n = 16), ACPA^-^ eRA (n = 18), and ACPA^+^ eRA (n = 19). The horizontal bar indicates the mean value. **(C)** Dot plot showing the serum concentration levels of interleukin-12 (IL-12) in UA (n = 21), controls (n = 16), ACPA^-^ eRA (n = 17), and ACPA^+^ eRA (n = 19). The horizontal bar represents the mean value. **(D)** Scatter plot illustrating the correlation between IFN-γ and IL-12 concentrations in serum samples. Statistical significance was assessed using the Kruskal–Wallis test for **(B, C)** and Pearson’s correlation coefficient for **(D)**. P-values less than 0.05 were considered significant.

To elucidate differences between ACPA^+^ eRA patients and ACPA^-^ eRA patients, we recruited 37 eRA patients, 16 unclassified arthritis patients, and 21 healthy participants. Based on the experimental design presented in **Figure 1A**, their serum cytokine levels and transcriptome were examined using MCA and scRNA-seq, respectively. Among eRA patients, the ACPA^+^ eRA group displayed elevated serum levels of IFN-γ and IL-12, the hallmark cytokines of type 2 interferon signaling, in comparison with the ACPA^-^ eRA group (**Figures 1B, C**). There was a strong positive correlation between the two cytokine levels (**Figure 1D**). These findings highlight that increased serum IFN-γ concentrations are closely related to the seropositivity of RA.

### Single-cell RNA-seq analysis landscape of eRA PBMCs

**Figure 2:**
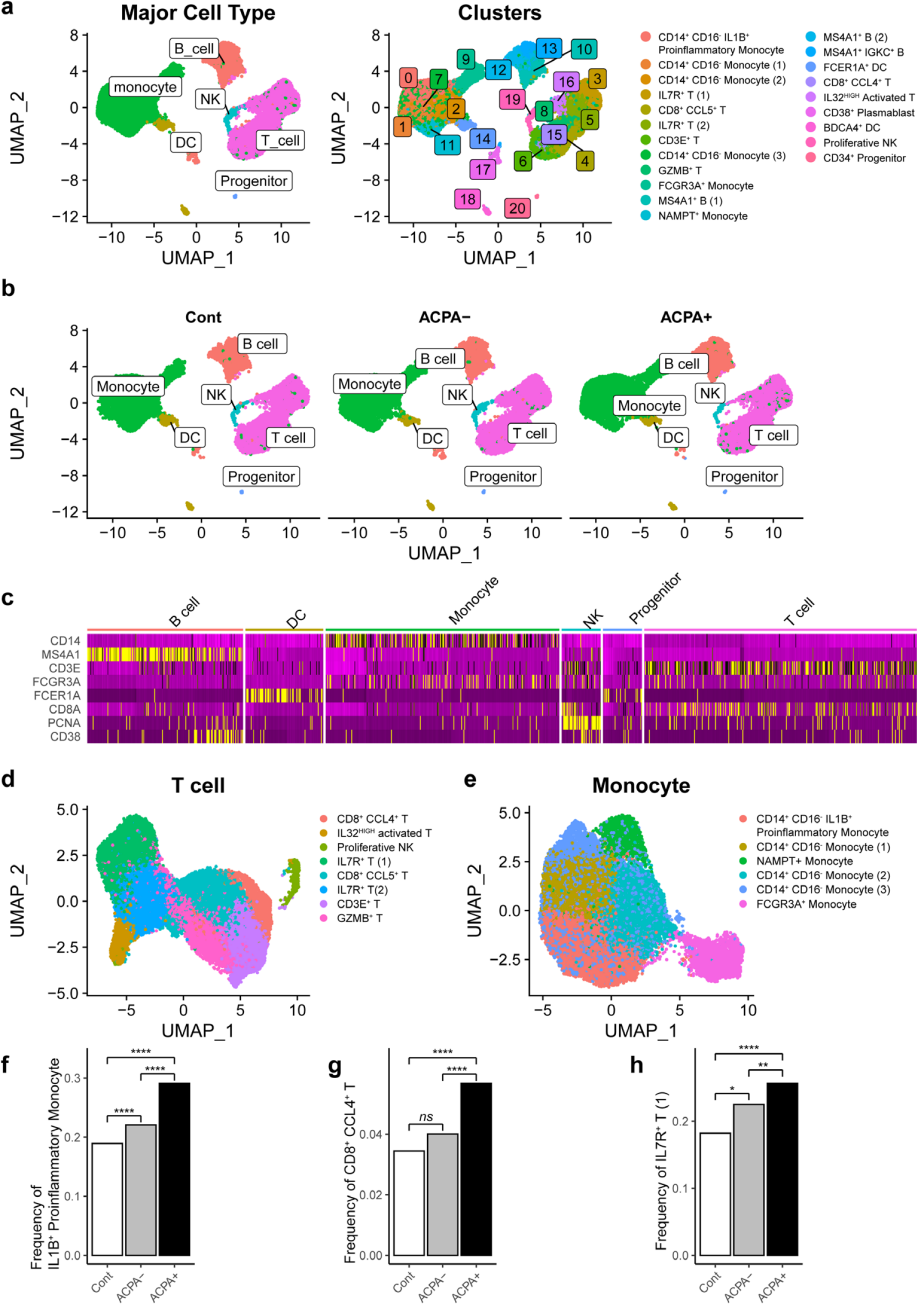
Cellular fractions of PBMCs reconstructed from single-cell RNA-sequencing (scRNA-seq) analysis in RA patients. **(A)** Uniform Manifold Approximation and Projection (UMAP) based on scRNA-seq data from PBMCs of drug naïve RA patients (n =12) and age-sex matched healthy controls (n = 4). A total of 37,318 cells were classified into 21 clusters (left panel) and differentiated into major cell types, including monocytes, dendritic cells (DCs), T cells, B cells, natural killer (NK) cells, and progenitors. **(B)** UMAP illustrates the distribution of the main cell types in PBMCs of control subjects, ACPA^-^ RA patients, and ACPA^+^ RA patients. **(C)** Heatmap representing marker gene expression patterns for major cell types across different clusters, providing a detailed view of gene expression signatures characteristic of each cell type. **(D, E)** Subsequent UMAP plots denoting sub-clusters and specific cell types for main cellular subsets, focusing on T cells **(D)** and monocytes **(E)**. **(F-H)** Bar graphs showcasing the relative proportion of specific cell subsets in PBMCs, including IL1B^+^ proinflammatory monocytes **(F)**, CD8^+^ CCL4^+^ T cells **(G)**, and IL7R^+^ T cells **(H)**, in control, ACPA^-^ RA, and ACPA^+^ RA group. P-values from z-tests for population proportions indicate significant cell type expansions. *P-value<0.05, **P-value<0.01, and ****P-value<0.0001.

We next conducted scRNA-seq to characterize and compare transcriptome profiles of CD45^+^ cells from PBMCs obtained from healthy controls (n = 4), drug-naïve ACPA^-^ eRA patients (n = 6), and drug-naïve ACPA^+^ eRA patients (n = 6). We initially addressed batch effects through a QC confirmation process (**Supplementary Figure S1**) and subsequently performed filtering procedures. A total of 37,318 immune cells were analyzed and segregated into 21 distinct clusters based on their transcriptomic profiles (**Figures 2A, B**). To identify the cell types within each cluster, we analyzed expression levels of marker genes (*CD14*, *MS4A1*, *CD3E*, *FCGR3A*, *FCER1A*, *CD8A*, *PCNA*, *CD38*, and *CD4*). The results are presented in **Figure 2C** and **Supplementary Figure S2A**. Those levels were cross-referenced with immune cell data predicted from singleR (**Supplementary Figure S2B**). Additionally, we consulted canonical cell marker expression (**Supplementary Figure 3**), Human Cell Atlas annotations, and gene sets extracted from publicly available scRNA-seq data on RA synovial cells (https://www.immport.org/shared/study/SDY998) (23) (**Supplementary Table S2**). As a result, we finally identified 17 unique cell types within 21 clusters.

A Uniform Manifold Approximation and Projection (UMAP) plot demonstrated six clusters for T cells, four clusters for monocytes, three clusters for B cells/plasmablasts, two clusters for dendritic cells, one cluster for natural killer (NK) cells, and one cluster for progenitor cells (**Figures 2A, D, E**). Proportions of B cells, dendritic cells, monocytes, and T cells were comparable between ACPA^-^ RA and ACPA^+^ eRA (**Supplementary Figure S2C**). However, we observed a substantial expansion of IL1B^+^ proinflammatory monocytes, CD8^+^ CCL4^+^ T cells, and IL7R^+^ T cells in eRA patients compared to healthy controls. Furthermore, these three subsets were significantly frequent in ACPA^+^ eRA than in ACPA^-^ eRA (**Figures 2F–H**).

Conclusively, through global transcriptome profiling, we identified 17 unique cell types in human PBMCs, including three subsets of immune cells, IL1B^+^ proinflammatory monocytes, CD8^+^ CCL4^+^ T cells, and IL7R^+^ T cells, presumably representing the peripheral landscape of immuno-pathology of ACPA^+^ eRA.

### Increased expression of interferon response genes in ACPA^+^ eRA

**Figure 3:**
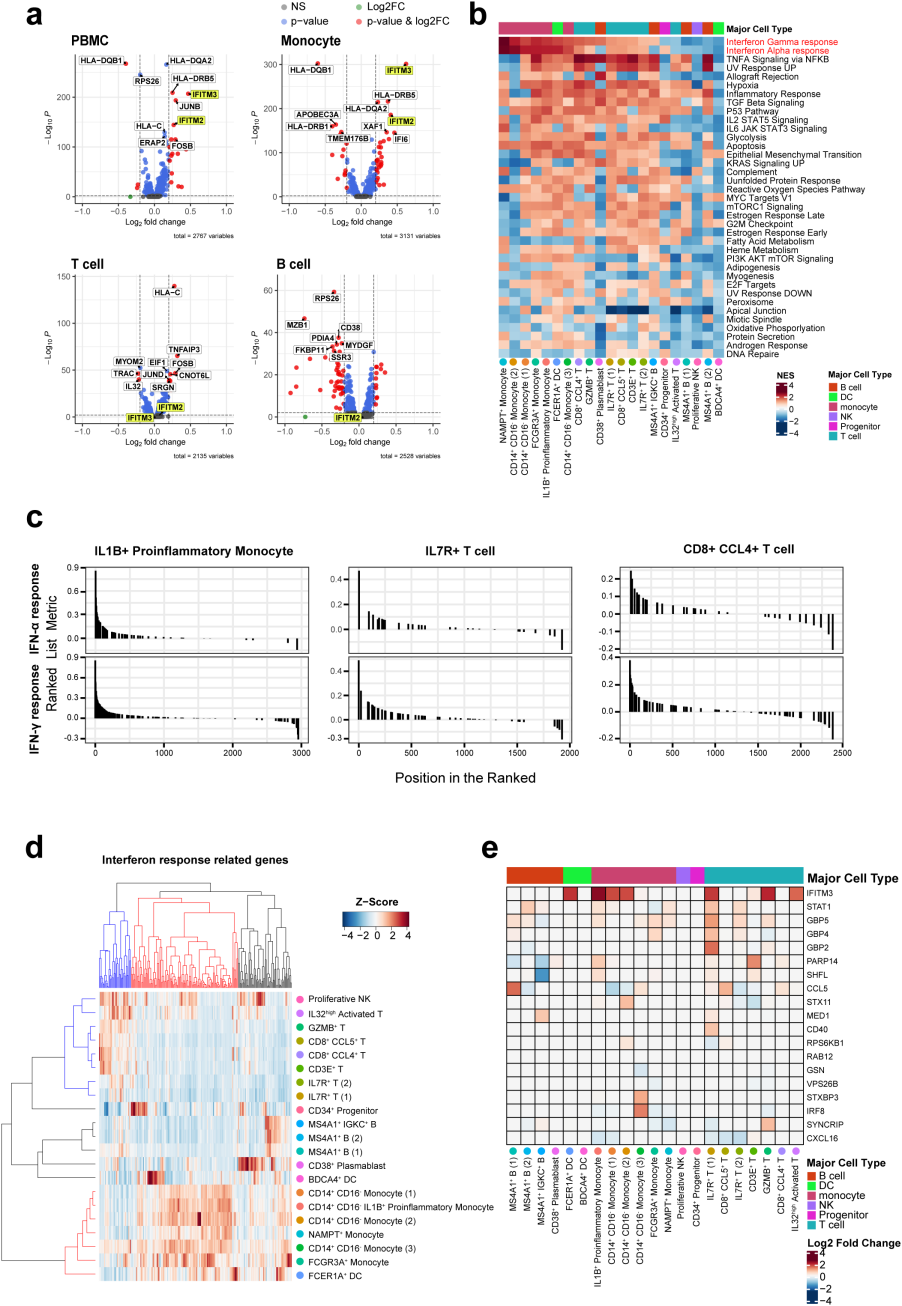
Differentially expressed gene sets in each cell subset based on gene ontology terms and Kyoto Encyclopedia of Genes and Genomes. **(A)** Volcano plot comparing ACPA^+^ and ACPA^-^ group for PBMCs, T cells, B cells, and monocytes, revealing differentially expressed genes. IFITM2/3 are highlighted in yellow. **(B)** Heat map displaying gene set enrichment analysis of genes with changing expression levels in ACPA^-^ and ACPA^+^ across 17 cell types. Column annotations on the heatmap show major cell types. Red color indicates the enrichment score increased in ACPA^+^ RA, and blue color indicates an increased enrichment score in ACPA^-^ RA. **(C)** GSEA plots of IFN-α and IFN-γ response gene sets in the three subsets expanded in ACPA^+^ patients, including IL1B^+^ proinflammatory monocytes, CD8^+^ CCL4^+^ T cells, and IL7R^+^ T cells. **(D, E)** Heatmap representing differences in expression of genes related to IFN-γ by cell type. The heatmap in **(D)** shows the expression patterns of IFN-α and IFN-γ response genes in each of the 21 cell clusters. In the dendrogram of both columns and rows, blue represents genes highly expressed in T cells, while red denotes those highly expressed in monocytes. The color matrix represents the Z-score of relative IRG expression levels within cells, where red indicates high expression and blue indicates low expression. The heatmap in **(E)** shows the log2 scaled fold change values of 19 IFN-γ response genes in ACPA^+^ patients compared to those in ACPA^-^ patients. The highly expressed genes in monocytes were selected for this heatmap. Red indicates an increase in ACPA^+^ patients, while blue indicates a decrease.

To gain a deeper understanding of the alterations in gene expression linked to ACPA positivity and to explore the underlying mechanisms of RA associated with these changes, we next analyzed DEGs in each cell type of PBMCs from ACPA^+^ and ACPA^-^ eRA patients. Given the multiplex cytokine data in **Figure 1**, we sought to focus on the IFN-γ and IL-12-JAK pathway for the analysis. The top 20 DEGs from the scRNA-seq analysis are listed, which included IRGs such as *IER3*, *JUNB*, and *IFITM2*, and *IFITM3*, and among them, *IFITM3* showed nearly the highest fold change (**Table 1**). Notably, volcano plots of cell subsets demonstrated that differential expression of *IFITM3* was mainly observed in monocytes and T cells, not in B cells (**Figure 3A**).

**Table 1:** The top 20 differentially expressed genes between ACPA^-^ and ACPA^+^ from PBMC scRNA-seq.

	*p*-value	avg_log2FC	pct.1	pct.2	*p*-value_(adj)
HLA-DQA2	7.97E-266	1.892949811	0.132	0.037	1.29E-261
IFITM3	1.79E-205	0.965439862	0.407	0.289	2.91E-201
HLA-DRB5	7.71E-191	0.729622267	0.313	0.189	1.25E-186
JUNB	6.23E-163	0.339508966	0.861	0.804	1.01E-158
IFITM2	6.14E-135	0.467551747	0.58	0.482	9.96E-131
ERAP2	3.73E-122	0.663834203	0.258	0.166	6.05E-118
BTG2	9.67E-112	0.405070841	0.605	0.519	1.57E-107
IFI44L	4.05E-110	0.903485471	0.179	0.105	6.57E-106
TNFSF10	3.71E-108	0.818914251	0.199	0.123	6.02E-104
IFI6	6.41E-108	0.789436412	0.311	0.227	1.04E-103
FOSB	7.27E-106	0.362939434	0.737	0.664	1.18E-101
IER3	1.07E-96	0.805034193	0.38	0.304	1.73E-92
XAF1	2.85E-96	0.612964022	0.313	0.23	4.62E-92
MNDA	4.82E-90	0.601017761	0.38	0.303	7.82E-86
HLA-C	6.54E-85	0.171143633	0.99	0.984	1.06E-80
FOS	1.16E-84	0.274752943	0.933	0.907	1.89E-80
TSC22D3	6.51E-84	0.370209625	0.56	0.481	1.06E-79
TNFAIP3	1.29E-79	0.383694023	0.657	0.589	2.09E-75
IER2	8.44E-75	0.358348283	0.567	0.498	1.37E-70
IFIT3	2.25E-73	1.163609645	0.075	0.035	3.64E-69

To compare functional characteristics of each cell subset between ACPA^+^ and ACPA^-^ eRA, we also performed Gene Set Enrichment Analysis (GSEA) using Hallmark gene sets provided by MsigDB. The results indicate that all monocyte subsets of ACPA^+^ eRA had higher transcriptional profiles for “IFN-γ response” and “IFN-α response” than those of ACPA^-^ eRA (**Figure 3B**). Most T cell subsets, with the exception of IL32^high^ T cells, exhibited strong enrichment for these IFN responses (**Figures 3B, C**). In particular, in ACPA^+^ eRA, the three cell subsets-IL1B^+^ proinflammatory monocytes, CD8^+^ CCL4^+^ T cells, and IL7R^+^ T cells-exhibited increased expansion with heightened enrichment profiles for IFN-γ and IFN-α responses compared to those in ACPA^-^ eRA (**Figure 3C**).

The hierarchical clustering and heat map analysis illustrate that upregulated IRGs in monocytes were different from those in T cells, indicating that transcriptional responses to IFN are different according to cell type (**Figures 3D, E**). Through pseudo-bulk analysis of scRNA-seq data, we also observed the increases in JAK-STAT pathway-related genes in IL7R^+^ T cells and IL1B^+^ proinflammatory monocytes of ACPA^+^ eRA patients as compared to those of ACPA^-^ eRA patients (**Supplementary Figures S4A, B, D, E**). Subsequent GSEA analysis revealed upregulation of the genes associated with the IL2-STAT5 signaling pathway (**Supplementary Figure S4C**). Given that IL-2 plays a role in Th1 differentiation by inducing the expression of IL-12 receptor and T-bet in a STAT5-dependent manner (30), this finding supports the notion that ACPA^+^ eRA has upregulated Th1 immunity. Interestingly, there was a strong positive correlation between ESR levels and IFN-γ signature genes in RA, which were obtained from the previously published data (**Supplementary Figures S5A, B**) (31–33). In summary, ACPA^+^ eRA showed increased activity of the IFN-JAK-STAT pathway as compared to ACPA^-^ eRA, which was more prominent in the cell types of IL1B^+^ proinflammatory monocytes and IL7R^+^ T cells.

### Upregulation of interferon-activated transcription factors in ACPA^+^ eRA

**Figure 4:**
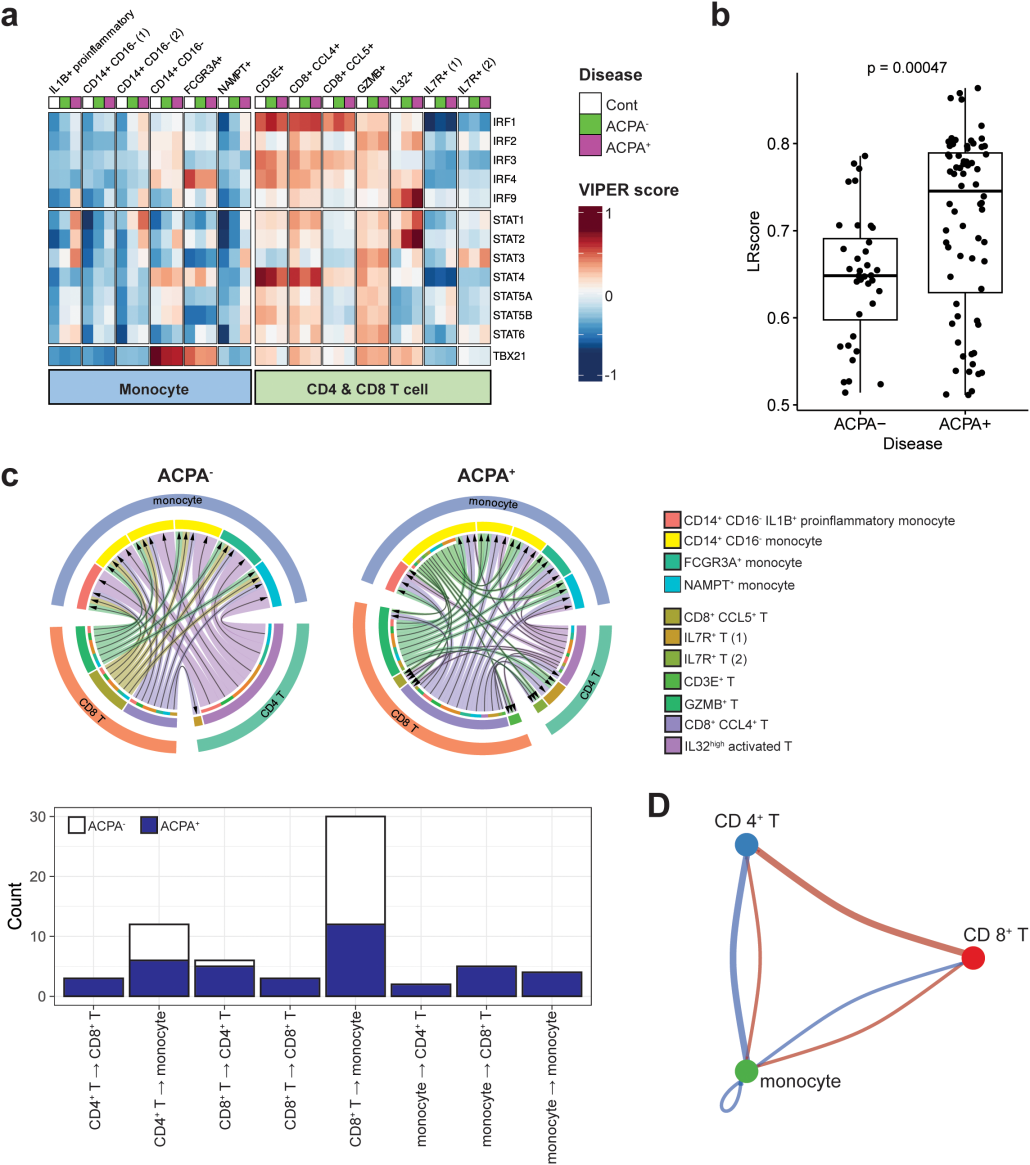
Downstream regulators of IFN-γ signaling and IFN-γ and its receptor interaction in monocytes and T cells of RA. **(A)** Heatmap displaying the activity scores of STAT and IRF family members predicted as downstream regulators of IFN-γ signaling. The scores were calculated by the DoRothEA package, a gene regulatory network containing signed transcription factor-target gene interactions. **(B)** Bar graph illustrating ligand-receptor (LR) scores for the interaction between IFN-γ (IFNG) and its receptor IFNGR1, derived from a ligand-receptor analysis. **(C)** Chord diagram illustrating variations in interactions between IFN-γ and IFNGR1 in monocytes and T cell subsets within ACPA^+^ and ACPA^-^ patient groups (upper panel). The outermost layer of the diagram represents the original major cell types, including monocytes, CD4+ T cells, and CD8+ T cells. Moving inward, the diagram shows the subsets of each cell type, which are color-coded according to the legend on the right side of the diagram. This color-coding is to facilitate the identification and differentiation of the various cell subsets within each primary cell category. The bar graph in the bottom panel depicts the directional variability of IFN-γ—IFN-γ receptor signaling in monocyte and T cell subsets from both ACPA^+^ and ACPA^-^ patients, showing the quantity of signals according to their signaling direction. **(D)** Cell-to-cell communication diagram depicts differential number of interactions between the three major cell types. Red indicates high in ACPA^+^ and blue indicates low in ACPA^-^.

IFN-γ is primarily produced by Th1 cells. It is a critical activator of immune response, promoting the killing of intracellular microbes by macrophages and dendritic cells (13, 34). In RA patients, IFN-γ is known to be produced due to Th1 skewing (14). Here, we focused on identifying TFs induced by IFN-γ signaling, including signal activators of transcription (STATs) and interferon regulatory factor (IRFs) (34, 35), in T cells and monocytes of PBMCs obtained from ACPA^+^ eRA versus ACPA^+^ eRA patients. To address this, we performed a master regulator analysis using VIPER score, which enabled us to identify differentially activated TFs in each cell type between healthy controls, ACPA^+^, and ACPA^-^ eRA. As a result, we found that IL1B^+^ proinflammatory monocytes and CD14^+^ CD16^-^ monocytes exhibited higher STAT1, 2, and 3 transcriptional activities in ACPA^+^ eRA patients than in ACPA^+^ eRA (**Figure 4A**). Moreover, IL1B^+^ proinflammatory monocytes, NAMPT^+^ monocytes, GZMB^+^ T cells, IL32^high^ T cells, and IL7R^+^ T cells in ACPA^+^ eRA exhibited higher STAT3 activity than other cell subsets (**Figure 4A**).

We further investigated the upstream reactions governing IRGs by quantitatively assessing the interaction of IFN-γ with its receptors using ligand-receptor (LR) scores. Our results indicate that the LR scores for IFN-γ-IFN-γ receptors were significantly higher in ACPA^+^ eRA than in ACPA^-^ eRA, as seen in the dot plot of LR scores above 0.5 (**Figure 4B**). Based on this interaction data, we next compared cell-to-cell interactions in ACPA^-^ eRA versus ACPA^+^ eRA, focusing on monocytes and T cell interaction. As shown in **Figure 4C**, the total number of IFN-γ-IFN-γ receptor interactions within diverse monocyte and T cell subsets was substantially higher in ACPA^+^ eRA than in ACPA^-^ eRA. Most strikingly, the interaction direction was entirely from T cells (→) to monocytes in ACPA^-^ eRA. In a sharp contrast, there were bidirectional and even multidirectional interactions between T cells and monocytes in ACPA^+^ eRA (**Figure 4C**, lower panel). Particularly, many interactions from monocytes (→) to T cells, in addition to those from T cells (→) to monocytes, were observed in ACPA^+^ eRA, and they were primarily driven by a subset of CD14^+^ CD16^-^ monocytes (*See* green arrows in the upper panel of **Figure 4C**). Moreover, we also detected interaction between CD4^+^ and CD8^+^ T cells, as well as between different subsets of monocytes in ACPA^+^ eRA, which were rarely found in ACPA^-^ eRA. To avoid any differences between the algorithms and sources of LR interaction information, we performed a cell-to-cell interaction analysis using CellPhoneDB (v.4.0) (27) and obtained the same results as previously obtained (**Supplementary Figure S6**). The cell-to-cell communications between CD4^+^, CD8^+^ T cells and monocytes were also detected in the analysis using CellChat (26) (**Figure 4D**).

Taken together, we observed the elevated STAT activity in IL1B^+^ proinflammatory monocytes in ACPA^+^ eRA, which seems to be associated with more interactions between IFN-γ-IFN-γ receptors. Notably, there were discernible patterns in monocyte and T cell interactions that appear to distinguish ACPA^+^ eRA (bidirectional) from ACPA^-^ eRA (unidirectional).

### Association of IFITM2/IFITM3 expression with STAT3, IL-6, and IFN-γ level in eRA patients

**Figure 5:**
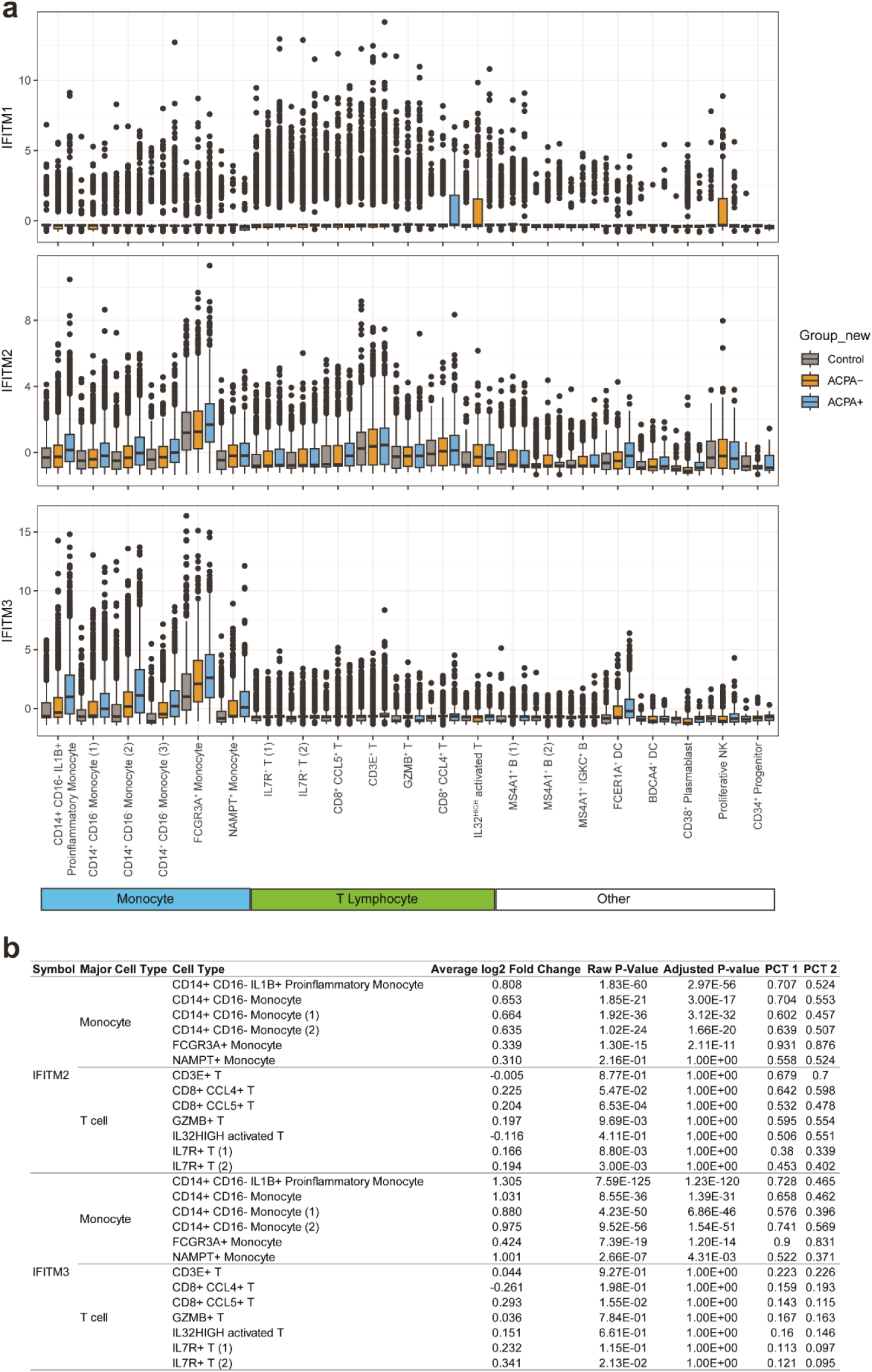
Comparison of expressions of *IFITM1*, *IFITM2*, and *IFITM3* across different cell types in the control, ACPA^-^ eRA, and ACPA^+^ eRA groups. **(A)** A boxplot showing the expression of *IFITM1*, *IFITM2*, and *IFITM3* in each cell type of control subjects, ACPA^-^ eRA patients, and ACPA^+^ eRA patients. **(B)** A table showing the differences in expression of *IFITM2* and *IFITM3* in monocytes and T cells, which was analyzed across control subjects, ACPA^-^ eRA patients, and ACPA^+^ eRA patients. The meanings of each statistical value are as follows: Average log2 Fold Change: The log fold-change of the average expression between the two groups. Positive value indicates a higher expression in ACPA^+^ eRA; Raw P-Value: The unadjusted *P*-value; Adjusted P-Value: The adjusted *P*-value, based on the Bonferroni correction using all features in the dataset. PCT1: The percentage of cells in which the feature is detected in the ACPA^+^ eRA; PCT2: The percentage of cells in which the feature is detected in the ACPA^-^ eRA.

**Figure 6:**
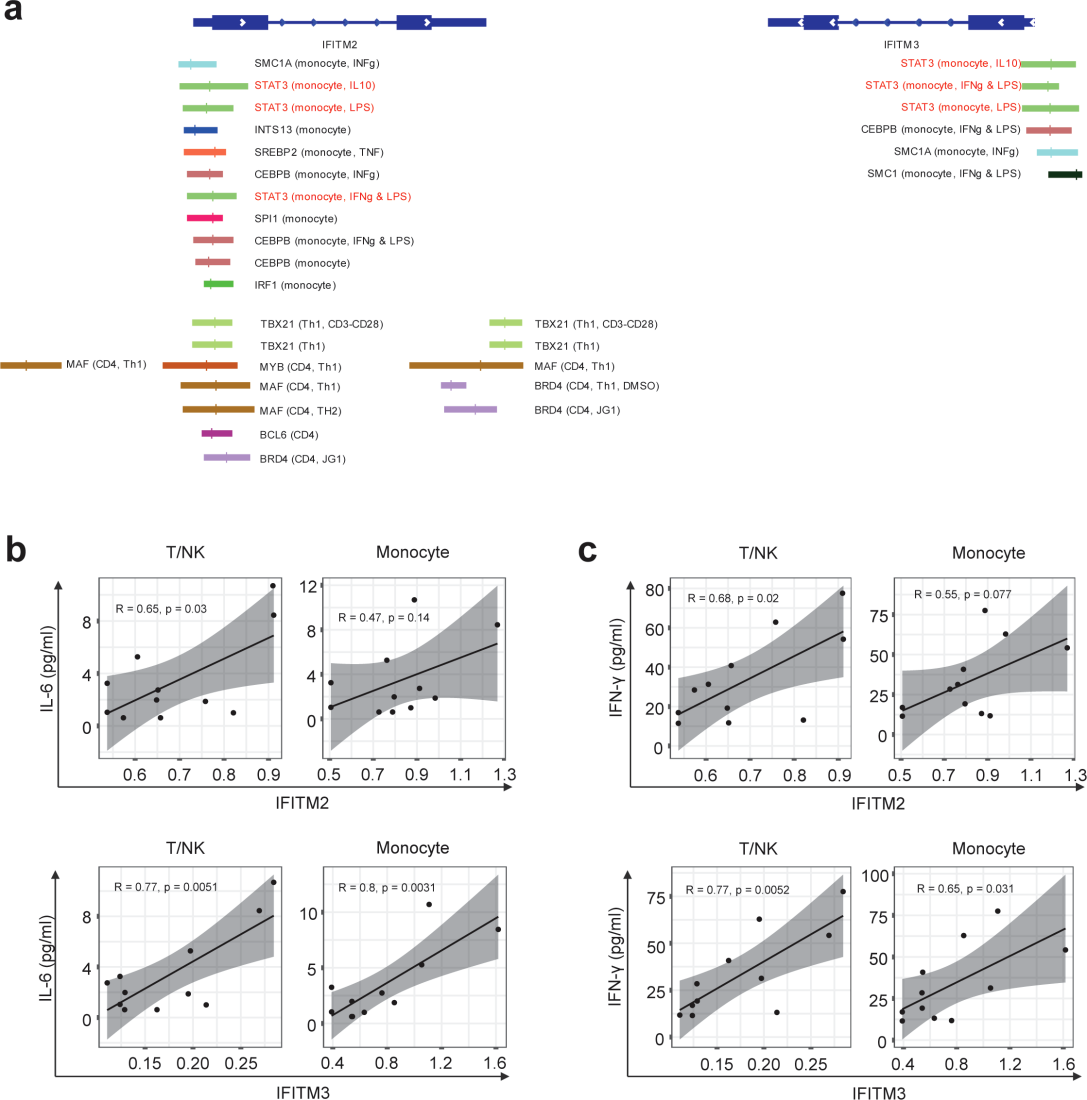
Differences in IFITM2/3 expression in monocytes and T cells of RA patients depending on the presence of anti-CCP antibodies. **(A)** Illustration depicting the transcription factors predicted to bind to the promoters of *IFITM2* and *IFITM3* genes, along with their specific binding locations. The binding sites for the same TF are indicated by the same color. The first parenthesis indicates which cell line it is from and the second indicates the treatment. The absence of these indicates the basal condition with no treatment. **(B, C)** Graphs correlating mRNA expression levels of *IFITM2* **(B)** and *IFITM3* **(C)** in T/NK cells and monocytes with the concentrations of IL-6 and interferons simultaneously measured in the serum of RA patients. The correlation coefficient (*R*) is indicated, and a linear regression line is fitted to the data points. Statistical significance of the correlation is assessed using Pearson’s correlation coefficient. P-values less than 0.05 were considered significant.

It is well known that IFITM2 and IFITM3 are induced by interferon stimulation (36, 37). As seen in **Table 1**, *IFITM2* and *IFITM3* were found to be the top 5 DEGs as IRGs (**Table 1**). Elevated levels of *IFITM2* and *IFITM3* were observed in the monocytes of ACPA^+^ eRA patients, especially in IL1B^+^ proinflammatory monocytes, which are presumably the M1 monocyte subset (**Figures 5A, B**). These findings have sparked our curiosity to explore further the regulatory mechanisms that control the transcription of IFITM2 and IFITM3 in ACPA^+^ eRA. To address this, we searched for the chromatin binding profiles of *IFITM2* and *IFITM3* (**Figure 6A**) using the ReMap2022 database (38). We identified 27 binding regions for 13 TFs from public data produced by 11 independent studies (**Supplementary Table S3**). The results showed that *STAT3*, which is known as an IFN-activated TF (39), was identified as one of the regulatory TFs for *IFITM2* and *IFITM3* transcription. These results, together with the data in **Figures 4A** and **B**, suggest that increased activation of STAT3 is involved in regulation of IFITM2 and IFITM3 in ACPA^+^ eRA.

Finally, we examined the relationships of *IFITM2* and *IFITM3* expression in monocytes and T cells with pathogenic cytokines of eRA (**Figures 6B, C**). We found a moderate positive correlation between serum IL-6 concentrations and expression levels of *IFITM2* in T/NK cells (*R* = 0.65, *p* = 0.03). The serum IL-6 level showed a strong positive association with expression levels of *IFITM3* in T/NK cells (*R* = 0.77, *p* = 0.0051) and *IFITM3* in monocytes (*R* = 0.8, *p* = 0.0031) (**Figure 6B**). Serum levels of IFN-γ had moderate to strong positive correlations with expression levels of *IFITM2* in T/NK cells (*R* = 0.68, *p* = 0.02), *IFITM3* in T/NK cells (*R* = 0.77, *p* = 0.0052), and *IFITM3* in monocytes (*R* = 0.65, *p* = 0.031) (**Figure 6C**).

Together, these data suggest that *IFITM2* and *IFITM3*, the highly upregulated IRGs in ACPA^+^ eRA, are associated with *STAT3* activation and increased serum levels of IL-6 and IFN-γ.

## Discussion

ACPA-based stratification is the most widely accepted method for classifying RA. Our study elaborated contrasting immunologic features depending on ACPA status by mainly investigating global transcriptome profile of RA PBMCs and serum cytokines. Here, we demonstrated that serum IFN-γ and IL-12 levels were higher in in ACPA^+^ eRA than in ACPA^-^ eRA and healthy controls, indicating a skewing towards a Th1 phenotype. Moreover, IL1B^+^ proinflammatory monocytes (most strikingly), CD8^+^ CCL4^+^ T cells, and IL7R^+^ T cells were expanded in ACPA^+^ eRA. In ACPA^+^ eRA, most of monocyte and T cell subsets, including the three expanded subsets of ACPA^+^ eRA, had upregulated expressions of IRGs related to IFN-γ and IFN-α. The observed elevation in the transcriptional activity of IRGs is supported by increased expression of STATs, the IFN-driven TFs, in monocytes of ACPA^+^ eRA. Notably, IFN-γ and its receptor interaction between monocytes and T cells in ACPA^+^ eRA was markedly increased and displayed a multi-directional pattern, contrasting with the unidirectional pattern observed in ACPA^-^ eRA. Together, these findings suggest that IFN-mediated responses are overactive in ACPA^+^ eRA. In support of this, *IFITM2* and *IFITM3* expression levels of in monocytes and T/NK cells had positive correlations with circulatory IL-6 and/or IFN-γ levels.

This study initially identified concurrent increases in serum levels of IFN-γ and IL-12, the Th1 cytokines, in ACPA^+^ eRA compared to ACPA^-^ eRA. Furthermore, our transcriptome analysis demonstrated an elevation of IRGs, suggesting substantial IFN-γ exposure in monocytes and T cells of ACPA^+^ eRA. Consistent with this, recent research shows that ACPA^+^ RA patients have a significantly higher count and proportion of circulating Th1 cells relative to ACPA^-^ RA patients (40). James et al. reported that Th1 cells are the most abundant subset within CD4^+^ T cells that are specific to citrullinated peptides in RA patients (41). Although we did not provide direct evidence of Th1 cell expansion in ACPA^+^ eRA cells, we observed an increase in the number of IL7R^+^ T cells. Notably, a large proportion of the IL7R^high^ CD62L^low^ T cells were Th1 cells (42). IL-7 potently stimulated IFN-γ production in synovial CD4^+^ T cells, suggesting a link between IL7R^+^ T cells and the Th1 response (43). Collectively, our findings not only support previous observations, but also underscore the critical role of Th1 immunity in the peripheral circulation of ACPA^+^ eRA through a systemic approach.

Here, we found that IFN-γ response genes were upregulated in ACPA^+^ eRA. IFN-γ both induces Th1 cell differentiation and is subsequently secreted by them, initiating a cascade of immunological responses (44). IFN-γ drives the polarization of macrophages into the M1 subtype and enhances antigen presentation via MHC class II (14). IFN-γ also induces synovial fibroblasts to express MHC class II, significantly enhancing their interaction with citrullinated vimentin upon autophagy induction (45). Beyond its association with autoimmunity, IFN-γ is involved with synovial inflammation in RA. An omics study revealed a notable expansion of HLA-DRA^hi^ sublining fibroblasts, enriched with *HLA-DR*, *HLA-DP*, and *IFN-γ-inducible protein 30*, in the leukocyte-rich RA synovium (23). IFN-activated monocytes, another highly expanded subset of these tissues, exhibit elevated IRG transcription (23). IFN-γ has a complex role, as mice lacking it are more prone to arthritis (46) and an anti-IFN-γ antibody was ineffective in an RA trial (14), yet excess levels are likely to exacerbate autoimmune diseases (14). This study suggests that IFN-γ response genes are enriched in ACPA^+^ eRA and may serve as a therapeutic target specific to this population, as earlier findings support that excess of IFN-γ is likely pathogenic.

Type I IFN, known for their antiviral role, also contribute to autoimmunity through maladaptive lymphocyte activation (47). Our scRNA-seq analysis of drug-naïve RA PBMCs suggested that type I IFN response genes were upregulated in monocytes and T cells of ACPA^+^ eRA. An earlier study supported this finding by showing a positive correlation between type I IFN signature gene expression and ACPA levels in patients with RA (31). Consistent with this, persistent stimulation of type I IFN can trigger the enhancement of T and B cell effector functions, resulting in the synthesis of autoantibodies (35, 47). IFN gene signatures and IFN-α levels are associated with RA disease activity (48). Additionally, IFN-activated monocytes are more abundant in leukocyte-rich synovial tissues compared to those with fewer leukocytes (23). Given the association of type I IFN with autoimmunity and RA severity, this study emphasizes the relevance of type I IFN in ACPA^+^ eRA and its potential as a therapeutic target.

According to the Accelerating Medicines Partnership (AMP) publication, bulk-RNA seq using leukocyte-rich RA synovium shows upregulation of IL1B and CCL4 in monocytes and CD8 T cells, respectively (23). IL1B is regarded as a conventionally important pathogenic cytokine of RA (2). Thus, increased IL1B^+^ monocytes support a more aggressive phenotype of ACPA^+^ eRA. CCL4, also known as macrophage inflammatory protein 1-β (MIP-1β), is amplified in the joint tissues and peripheral circulation of patients with RA. This amplification of CCL4 facilitates the migration of inflammatory cells and osteoclasts, positioning it as a significant pathogenic chemokine in RA (49). Our scRNA-seq data on the higher proportion of IL1B^+^ proinflammatory monocytes and CD8^+^ CCL4^+^ T cells in ACPA^+^ eRA are compatible to the previous reports (23), which indicates that the peripheral scRNA-seq landscape may be a molecular reflection of immunologic dysregulation in synovial compartment of RA patients, suggesting a possible communication between the periphery and the joints in establishing RA pathology.

Of note, our scRNA-seq analysis revealed that *IFITM2* and *IFITM3* belonging to IRGs were included in the top 20 DEGs. The human genome encodes at least five IFITM proteins. In particular, IFITM1, IFITM2, and IFITM3 have antiviral activities by inhibiting viral entry into human cells and some other pathways (37). Both type I and II IFNs can increase the expression of IFITM1, IFITM2, and IFITM3. In mice, among those IFITM proteins, *Ifitm3* is most strongly induced by IFN (37). Despite the proven role of IFITMs in defense against viral infection and in the mouse system, little is known about their role in human RA. *IFITM3* is one of the marker genes of the IFN-activated monocyte subset in synovial tissues; however, how the expression of *IFITM3* affects RA pathogenesis remains unclear (23). Here, we found that expression levels of *IFITM3* and *IFITM2* in monocytes and T/NK cells had a positive linear relation with the serum levels of IL-6 and/or IFN-γ, suggesting a possible induction of *IFITM2* and *IFITM3* by a cytokine-rich microenvironment. Notably, the specific linkage of IFITMs with IFN-γ points to a possible role for IFITMs in influencing Th1 skewing in the immunological dynamics of ACPA^+^ eRA. This finding is particularly intriguing as it offers a new perspective on the functions of IFITMs, extending beyond their established antiviral roles. Given its promise, this hypothesis warrants further detailed exploration.

In sum, our data, along with the earlier studies, indicate that targeting type 1 and 2 IFN signaling may be a patient-centric approach for ACPA^+^ RA patients. In this regard, the JAK inhibitor, which targets type I and type II IFNs (18), can be more effective in treating ACPA^+^ eRA than ACPA^-^ eRA. In support of this notion, two clinical studies have shown that ACPA positivity leads to a higher rate of drug retention of JAK inhibitor, an indicator for overall effectiveness and safety of a drug (9, 50). Although earlier findings suggest that an IFN-targeting strategy may hold promise for better efficacy in patients with ACPA^+^ RA, further research is needed to confirm its superiority in this population.

This study has several limitations. First, we did not perform experimental validation of the findings from the scRNA-seq analysis. Second, the sample size for scRNA-seq analysis was small. Third, this study provides only an immunological basis that supports the potential for greater efficacy of interferon-targeting strategies in ACPA^+^ eRA than in ACPA^-^ eRA, which was not examined here. Therefore, these findings require further confirmation in follow-up studies.

To summarize, we observed differences in cytokine profiles, cell subset abundance, and gene expression patterns within the peripheral landscape between ACPA^+^ eRA and ACPA^-^ eRA. In ACPA^+^ eRA, serum IFN-γ levels were elevated, and peripheral blood T cells and monocytes exhibited upregulated IFN-γ response genes and IFN-γ-mediated cell-cell interactions, suggesting Th1 skewing. Moreover, ACPA^+^ eRA patients showed an expanded population of IL1B^+^ proinflammatory monocytes, CD8^+^ CCL4^+^ T cells, and IL7R^+^ T cells, in which IRGs were upregulated. Particularly, IFITM 2 and 3, which are associated with IRGs, could be new biomarkers for ACPA^+^ RA, offering promising avenues for future research and treatment strategies in eRA.

## Data Availability

The datasets presented in this study can be found in online repositories. The names of the repository/repositories and accession number(s) can be found below: GSE260796 (GEO).
