# Supplementary material for: Upregulation of interferon-γ response genes in monocytes and T cells identified by single-cell transcriptomics in patients with anti-citrullinated peptide antibody-positive early rheumatoid arthritis
Source: Front Immunol. 2025 Jan 14;15:1439082. doi: 10.3389/fimmu.2024.1439082 (PMC11772891; doi:10.3389/fimmu.2024.1439082)
Supplement: Supplementary file 1 [file DataSheet1.docx]

Supplementary Material

**Supplementary Figure 1.** Characterization of PBMCs in scRNA-seq analysis: patient data, batch effects, and cell type distribution.

**Supplementary Figure 2.** Detailed cellular landscape and cellular composition of PBMCs according to the presence of ACPA.

**Supplementary Figure 3.** Feature plots for marker genes that distinguish sub-clusters within primary cell subsets of B cells (A), T/NK cells (B), and monocyte/DC (C).

**Supplementary Figure 4.** Pseudo-bulk analysis results for IL7R^+^ T cells and IL1B^+^ proinflammatory monocytes.

**Supplementary Figure 5.** Correlation between ESR and interferon signature score.

**Supplementary Figure 6.** CellPhoneDB analysis of IFN-γ and its receptor interactions (A) and communications of monocytes and T cells (B).

**Supplementary Table 1.** Demographics and patient characteristics at baseline.

**Supplementary Table 2.** Results of cell type annotations.

**Supplementary Table 3**. List of Transcription Factors predicted to bind on IFITM2/3 5'-UTR.

**
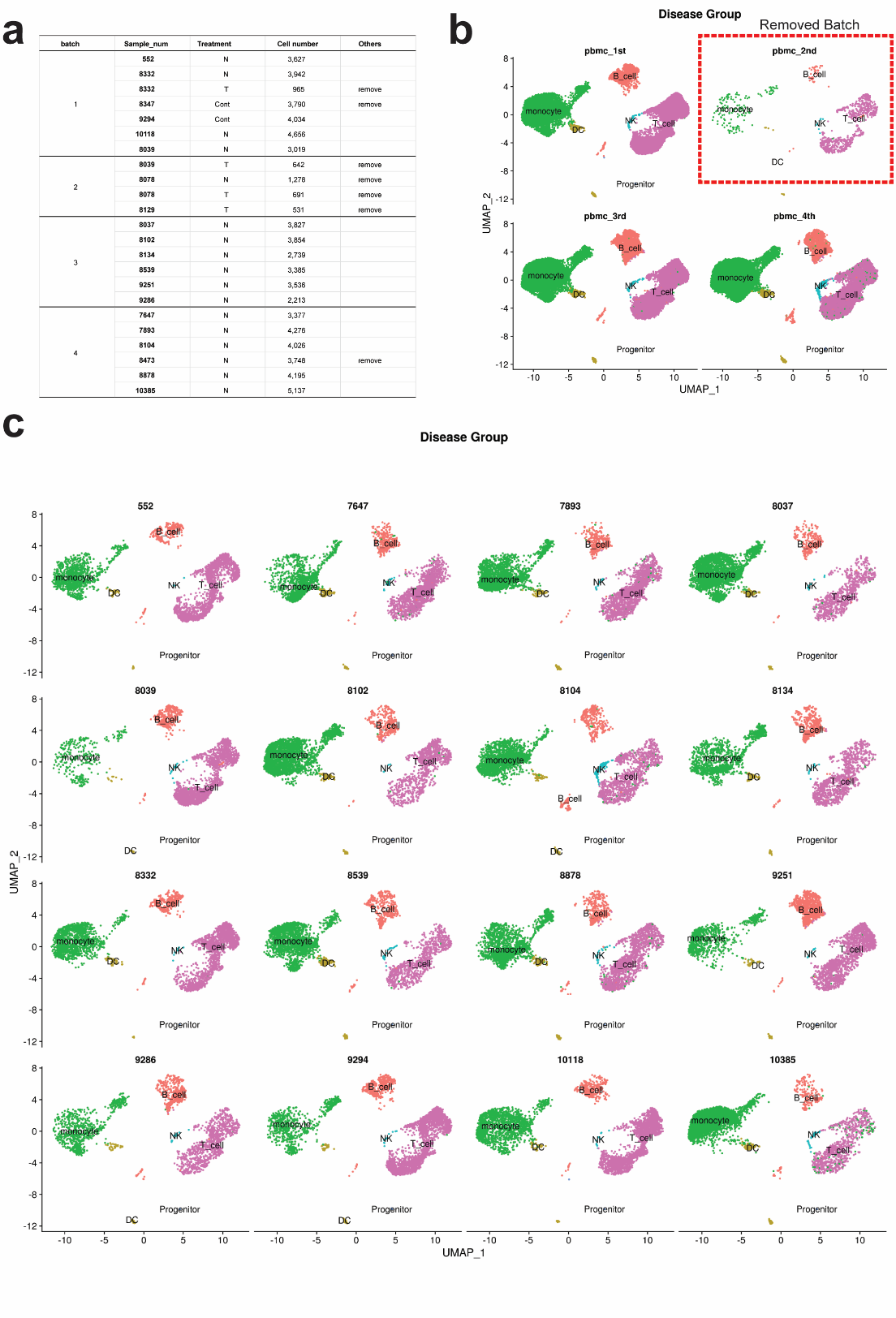
**

**Supplementary Figure 1. Characterization of PBMCs in scRNA-seq analysis: patient data, batch effects, and cell type distribution.** **(A)** A comprehensive table presenting detailed information on patients whose samples were used to create single-cell RNA sequencing (scRNA-seq) libraries. **(B)** UMAP visualization illustrates the segregation of data according to individual batch processing. A red box indicates the batch that has been excluded from the analysis. **(C)** UMAP plot showcasing the major cell types identified in peripheral blood mononuclear cells (PBMCs) after quality assessment, with each cell type distinguished according to the patient of origin.


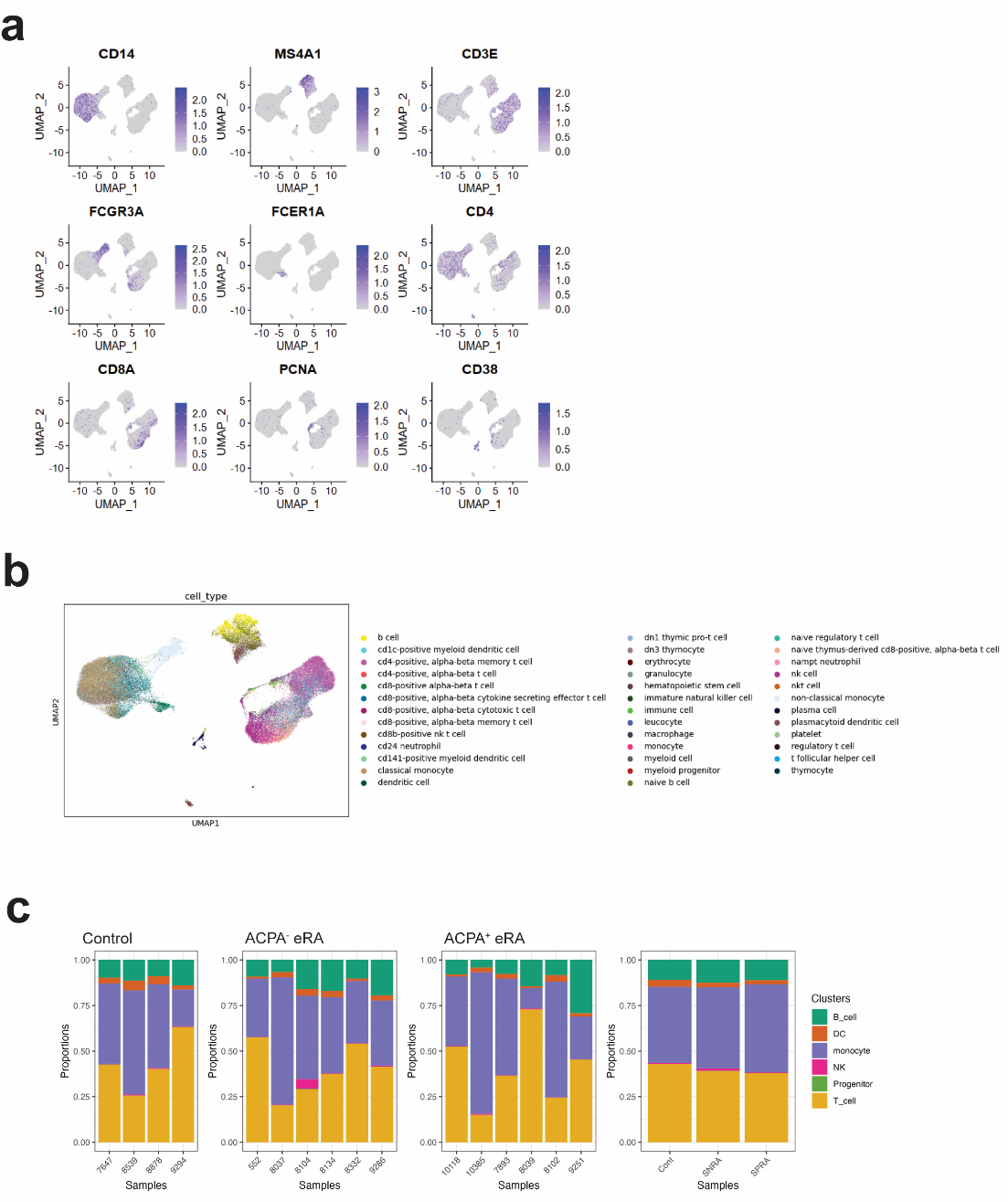


**Supplementary Figure 2**. **Detailed cellular landscape and cellular composition of PBMCs according to the presence of ACPA.** **(A)** Feature plots showcasing nine canonical marker genes (CD14, CD3E, CD4, CD8A, MS4A1, FCGR3A, FCER1A, PCNA, and CD38) expression across different cell clusters. **(B)** UMAP representation of cell type annotations based on SingleR analysis. **(C)** Bar graphs presenting a comparative analysis of the proportions of major immune cell types, including B cells, dendritic cells (DCs), monocytes, natural killer (NK) cells, progenitor cells, and T cells, across individual patients or grouped into control, ACPA^-^, and ACPA^+^ groups.


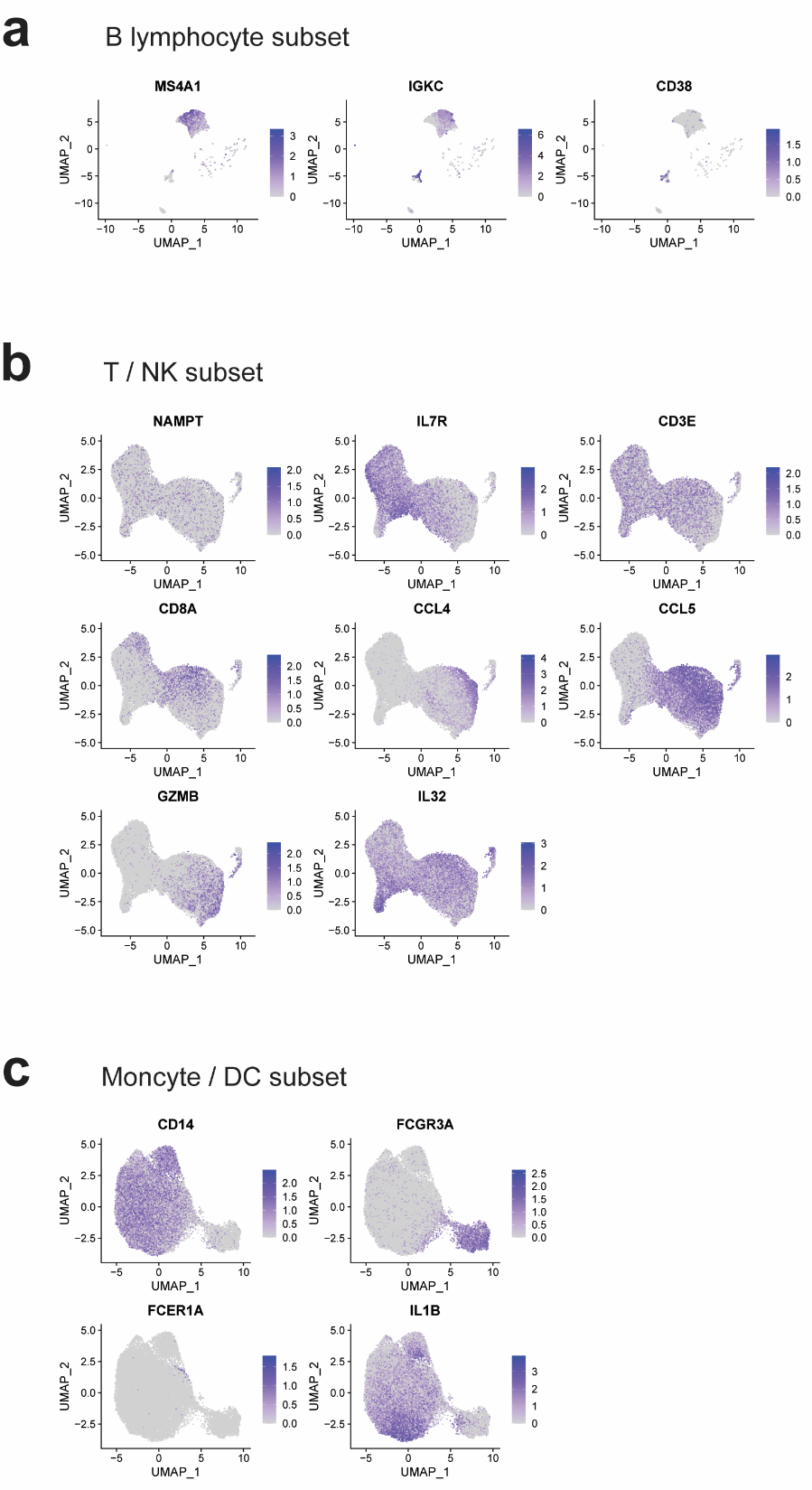


**Supplementary Figure 3. Feature plots for marker genes that distinguish sub-clusters within primary cell subsets of B cells (A), T/NK cells (B), and monocyte/DC (C).**


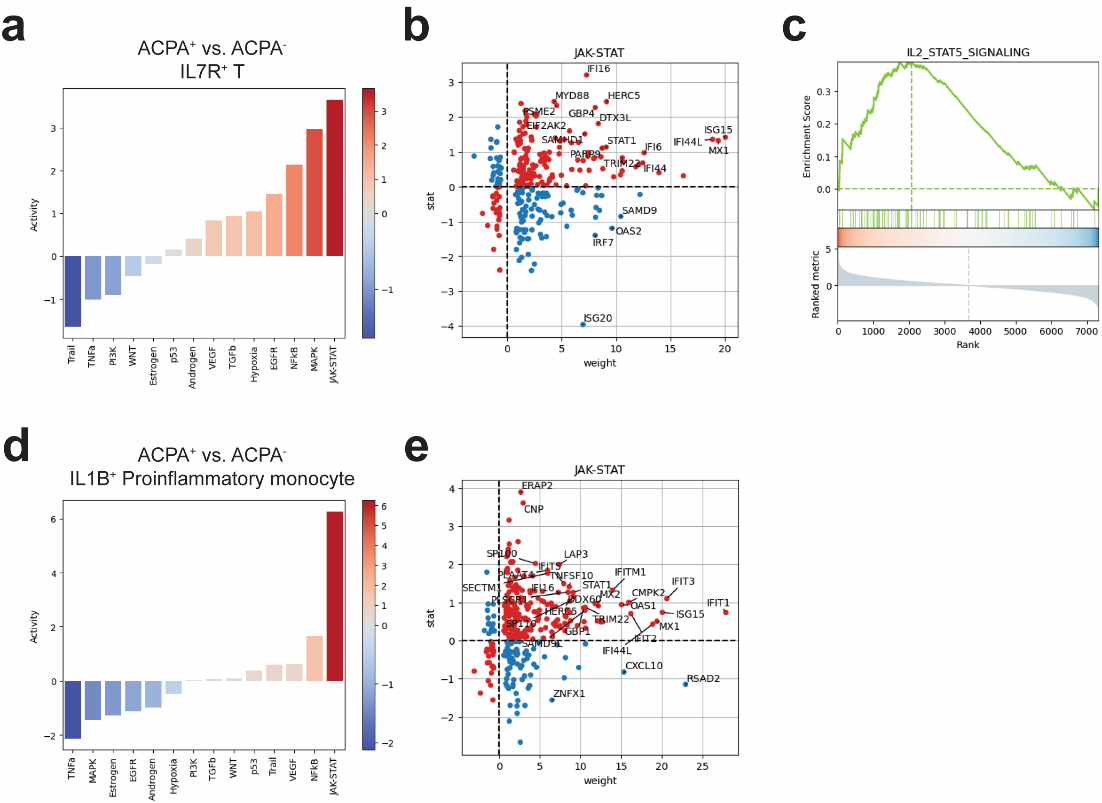


**Supplementary Figure 4. Pseudo-bulk analysis results for IL7R^+^ T cells and IL1B^+^ proinflammatory monocytes. (A)** Bar plot depicting pathway enrichment scores in IL7R^+^ T cells of ACPA^+^ RA patients compared to those of ACPA^-^ RA patients as inferred by the Multivariate Linear Model (MLM) of decoupleR. **(B)** Scatter plot illustrating the activity of target genes of the JAK-STAT pathway in IL7R^+^ T cells of ACPA^+^ RA patients compared to ACPA^-^ RA patients. **(C)** Results from Over Representation Analysis (ORA) conducted to identify significantly enriched pathways and biological processes in IL7R^+^ T cells of ACPA^+^ RA patients compared to those of ACPA^-^ RA patients. **(D)** Bar plot depicting pathway enrichment scores in IL1B^+^ proinflammatory monocytes from ACPA^+^ RA patients as compared to those from ACPA^-^ RA patients. **(E)** Scatter plot illustrating the activity of target genes of the JAK-STAT pathway in IL1B^+^ proinflammatory monocytes of ACPA^+^ RA patients compared to those of ACPA^-^ RA patients.


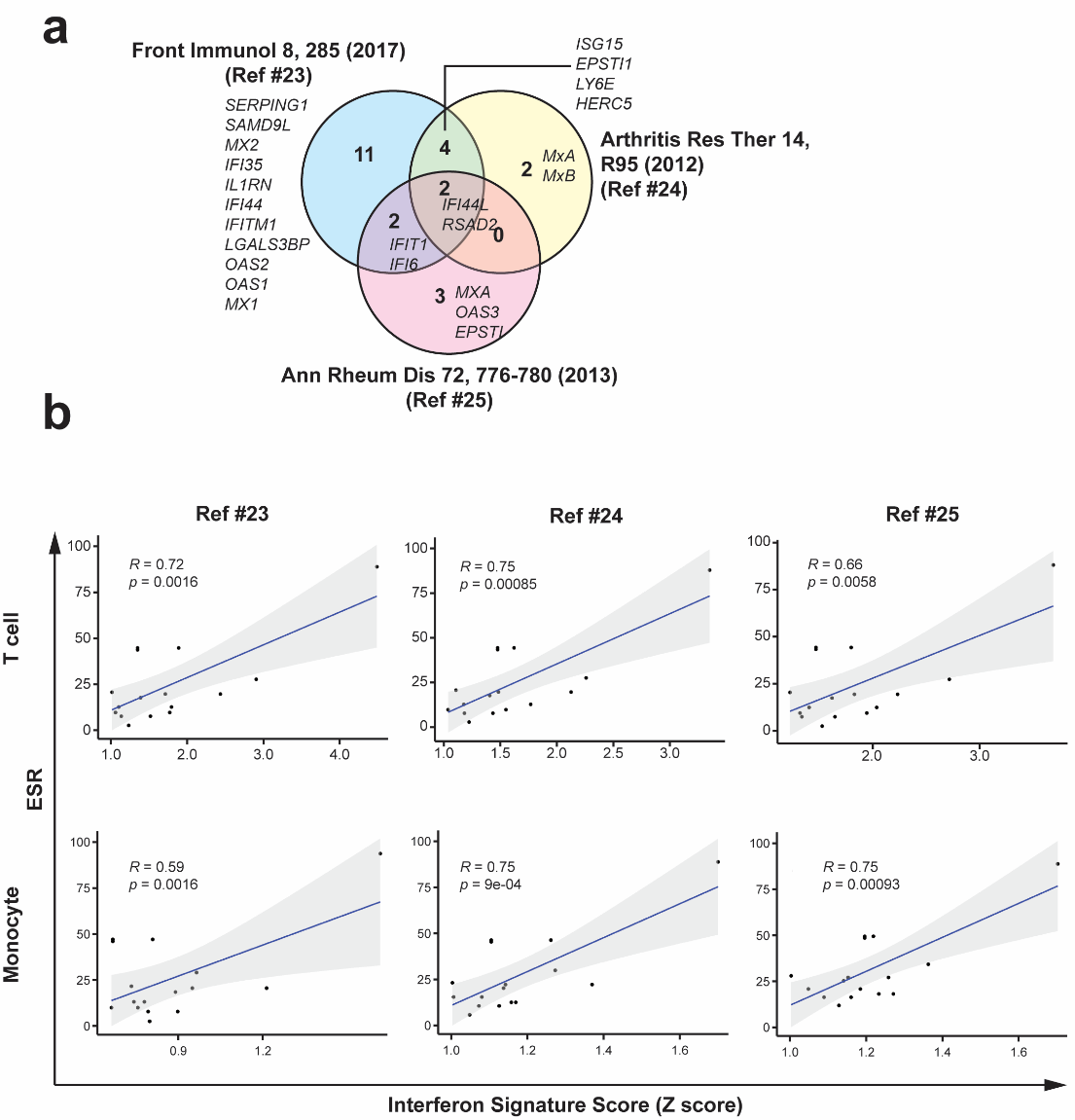


**Supplementary Figure 5. Correlation between ESR and interferon signature score.** **(A)** Venn diagram illustrating three gene sets for interferon signature score obtained from previous research. **(B)** Scatter plots showing correlations between patient ESR and interferon signature scores.


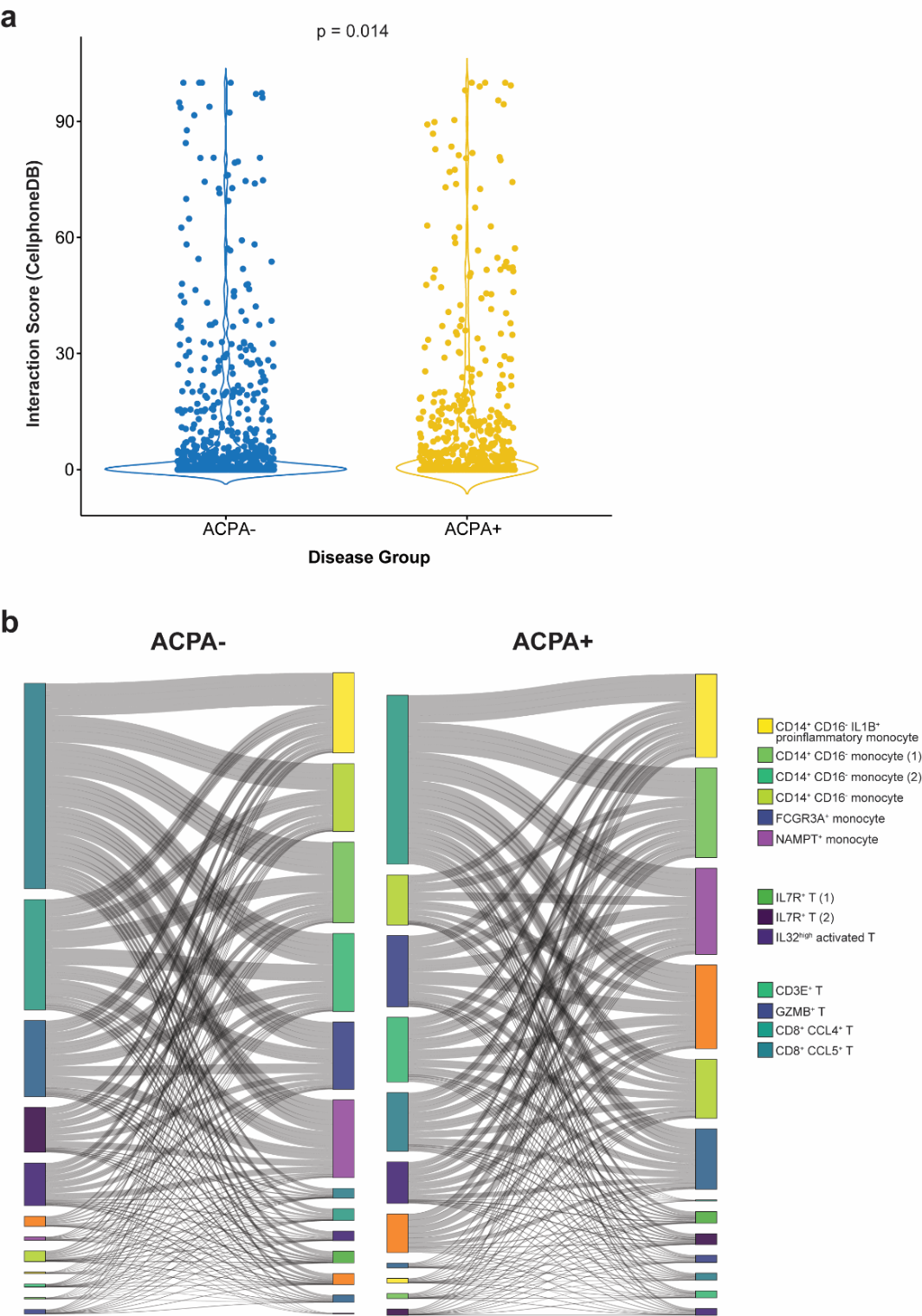
**Supplementary Figure 6. CellPhoneDB analysis of IFN-γ and its receptor interactions (A) and communications of monocytes and T cells (B).**

|  | **Unclassified  arthritis** | **Control** | **ACPA^-^ eRA** | **ACPA^+^ eRA** |
| --- | --- | --- | --- | --- |
| **Number** | 21 | 16 | 18 | 19 |
| **Age, years** | 56 ± 15 | 37 ± 7 | 34 ± 10 | 46 ± 12 |
| **Female** | 16 (76%) | 13 (81%) | 10 (56%) | 16 (84%) |
| **ESR, mm/hour** | 16.2 ± 12.5 | 10.6 ± 4.7 | 33.0 ± 26.7 | 30.2 ± 25.4 |
| **CRP, mg/L** | 6.7 ± 17.6 | 0.4 ± 0.3 | 18.4 ± 33.5 | 13.2 ± 27.3 |
| **RF positive** | 4 (19%) | - | 0 (0%) | 15 (79%) |
| **ACPA positive** | 0 (0%) | - | 0 (0%) | 19 (100%) |
| **DAS28-ESR** | - | - | 4.5 ± 1.0 | 4.9 ± 0.7 |
| **DAS28-CRP** | - | - | 4.0 ± 0.9 | 4.3 ± 0.6 |

**Supplementary Table 1.** Demographics and patient characteristics at baseline.

Data are presented as the mean ± SD or number (%).

*ACPA* anti-citrullinated peptide antibody, *RA* rheumatoid arthritis, *ESR* erythrocyte sedimentation rate, *CRP* C-reactive protein, *RF* rheumatoid factor, *DAS28* 28-joint disease activity score.

**Supplementary Table 2.** Results of cell type annotations.

| **Major Cell Type** | **Seurat annotation** | **Marker** | **Human Cell Atlas** | **Z annotation** | **Seurat**  **clusters** |
| --- | --- | --- | --- | --- | --- |
| **B** | MS4A1+ B | MS4A1 | CD19+ B cells (neg. sel) | - | 10, 12 |
|  | IGKC+ MS4A1+ Plasma cell | MS4A1, IGKC | CD19+ B cells (neg. sel) | - | 13 |
|  | IGKC+ CD38 high circulating Plasmablasts | IGKC, CD38 | Plasma cell | SC-B4 (Plasmablasts) | 17 |
| **T** | CCL5+ CD8+ T | CD3E, CD8, CCL5 | CD8+ T | - | 4 |
| **(CTL)** | GNLY+ GZMB+ T | CD3E, GNLY, GZMB | CD56+ NK | - | 6 |
|  | GNLY+ T | GNLY | CD56+ NK | - | 8 |
|  | CCL4+ CD8+ memory T | CD8, CCL4 | CD56+ NK | - | 15 |
| **DC** | FCER1A+ DC | FCER1A | DC | - | 14 |
|  | IRF8+ IRF4+ B | IRF4, IRF8 | BDCA4+ DC | - | 18 |
| **Monocyte** | IL1B+ proinflammatory  macrophage | CD14 | CD33+ Myeloid | SC-M1 | 0, 1, 2, 7, 11 |
| **NK** | FCGR3A+ NK | FCGR3A | CD14+ Monocyte | - | 9 |
|  | PCNA+ Proliferative | PCNA | 721 B  lymphoblasts | - | 19 |
| **Progenitor** | CD34+ progenitor | CD34 |  | - | 20 |
| **T** | IL7R+ T | CD3E, IL7R | CD4+ T | - | 3, 5 |
|  | IL32 high activated T | IL32 |  | SC-T2 (Treg) | 16 |

**Supplementary Table 3**. List of Transcription Factors predicted to bind on IFITM2/3 5'-UTR.

| **Target** | **GSE  accession ID** | **Transcription  Factor** | **Source Cell** |  | **Cell  Condition** | **Position** | | |
| --- | --- | --- | --- | --- | --- | --- | --- | --- |
|  |  |  |  |  |  | **Chr** | **Start** | **End** |
| IFITM2 | GSE59933 | BCL6 | CD4 |  |  | chr11 | 308145 | 308279 |
|  | GSE33281 | BRD4 | CD4 (JG1) |  |  | chr11 | 308155 | 308358 |
|  |  | BRD4 | CD4 (JG1) |  |  | chr11 | 309224 | 309454 |
|  | GSE62482 | BRD4 | CD4 (Th1) |  | DMSO | chr11 | 309207 | 309317 |
|  | GSE72266 | MAF | CD4 (Th1) |  |  | chr11 | 307251 | 307519 |
|  |  |  |  |  |  | chr11 | 308053 | 308357 |
|  |  |  |  |  |  | chr11 | 309067 | 309571 |
|  |  |  | CD4 (Th2) |  |  | chr11 | 308060 | 308377 |
|  |  | MYB | CD4 (Th1) |  |  | chr11 | 307971 | 308302 |
|  | GSE100381 | IRF1 | monocyte |  | No treatment | chr11 | 308152 | 308283 |
|  | GSE106359 | INTS13 | monocyte |  |  | chr11 | 308063 | 308216 |
|  | GSE120943 | CEBPB | monocyte |  | IFN-γ | chr11 | 308077 | 308237 |
|  |  |  |  |  | IFN-γ-LPS | chr11 | 308106 | 308282 |
|  |  | STAT3 | monocyte |  | IFN-γ-LPS | chr11 | 308079 | 308297 |
|  |  |  |  |  | IL10 | chr11 | 308045 | 308352 |
|  |  |  |  |  | LPS | chr11 | 308062 | 308284 |
|  | GSE129202 | SREBP2 | monocyte |  | TNF | chr11 | 308064 | 308250 |
|  | GSE31621 | CEBPB | monocyte |  |  | chr11 | 308116 | 308269 |
|  |  | SPI1 | monocyte |  |  | chr11 | 308079 | 308238 |
|  | GSE98367 | SMC1A | monocyte |  | IFN-γ | chr11 | 308041 | 308210 |
|  | GSE81881 | TBX21 | Th1 |  | CD3-CD28 (donor2) | chr11 | 308103 | 308279 |
| IFITM3 | GSE120943 | CEBPB | monocyte |  | IFN-γ-LPS | chr11 | 320826 | 321026 |
|  |  | SMC1 | monocyte |  | IFN-γ-LPS | chr11 | 320923 | 321072 |
|  |  | STAT3 | monocyte |  | IFN-γ-LPS | chr11 | 320805 | 320970 |
|  |  |  |  |  | IL10 | chr11 | 320799 | 321043 |
|  |  |  |  |  | LPS | chr11 | 320806 | 321059 |
|  | GSE98367 | SMC1A | monocyte |  | IFN-γ | chr11 | 320870 | 321052 |
